# Grain Size and Electrochemical Surface Modification Effects on Corrosion, Biological, and Technological Properties of CP Titanium Implants

**DOI:** 10.3390/jfb16120439

**Published:** 2025-11-25

**Authors:** Josef Hlinka, Daniel Cvejn, Ludek Dluhos, Vaclav Babuska, Kristina Cabanova, Jana Dvorakova, Anastasia Volodarskaja, Ruslan Z. Valiev, Nadimul H. Faisal, Katerina Peterek Dedkova, Renata Palupcikova, Vlastimil Vodarek

**Affiliations:** 1 Department of Materials Engineering and Recycling, Faculty of Materials and Technology, VSB-Technical University of Ostrava, 17. listopadu 2172/15, 708 00 Ostrava-Poruba, Czech Republic; Anastasia.volodarskaja@vsb.cz (A.V.); renata.palupcikova@vsb.cz (R.P.); vlastimil.vodarek@vsb.cz (V.V.); 2 Centre for Advanced Innovation Technologies, VSB-Technical University of Ostrava, 17. listopadu 2172/15, 708 00 Ostrava-Poruba, Czech Republic; daniel.cvejn@vsb.cz; 3 TIMPLANT, Ltd., Sjednoceni 77/1, Polanka nad Odrou, 725 25 Ostrava-Poruba, Czech Republic; timplant@timplant.cz; 4 Department of Medical Chemistry and Biochemistry, Faculty of Medicine in Pilsen, Charles University, Alej Svobody 76, 323 00 Pilsen, Czech Republic; vaclav.babuska@lfp.cuni.cz (V.B.); jana.dvorakova@lfp.cuni.cz (J.D.); 5 Department of Mining Engineering and Safety, Faculty of Mining and Geology, VSB-Technical University of Ostrava, 17. listopadu 2172/15, 708 00 Ostrava-Poruba, Czech Republic; kristina.cabanova@vsb.cz (K.C.); katerina.peterek.dedkova@vsb.cz (K.P.D.); 6 Institute of Physics of Advanced Materials, Ufa University of Science and Technology, Ufa 450076, Russia; ruslan.valiev@ugatu.su; 7 Liaoning Academy of Materials, 280, Chuangxin Road, Hunnan District, Shenyang 110167, China; 8 School of Computing, Engineering and Technology, Robert Gordon University, Garthdee Road, Aberdeen AB10 7GJ, UK; n.h.faisal@rgu.ac.uk

**Keywords:** titanium implants, nanostructure, corrosion, biocompatibility, surface treatment, targeted drug delivery system

## Abstract

Commercially pure (CP) titanium is widely used for long-term biomedical implants due to its high biocompatibility and corrosion resistance. However, its relatively low strength limits its use in highly loaded applications. Ultrafine-grained (UFG) titanium obtained through severe plastic deformation offers enhanced mechanical performance while maintaining the stability of CP titanium. This study investigates how electrochemical surface modification by anodization affects the corrosion, biological performance, and technological behavior of UFG titanium. TiO_2_ layers with nanotubular and nanoporous morphologies were produced at anodization voltages between 20 and 60 V. Corrosion tests in physiological solution confirmed stable passive behavior with corrosion rates below 4 µm year^−1^, and surface wettability increased markedly with anodization. Osteoblast-like MG-63 cells exhibited good viability on all anodized surfaces, with improved adhesion and proliferation on samples anodized at 60 V. The porous TiO_2_ layers were successfully intercalated with dimethyl sulfoxide and ibuprofen, demonstrating potential for local drug delivery. Implantation simulations on real Nanoimplant^®^ prototypes confirmed sufficient mechanical stability of the anodized layer. Overall, the optimized anodization of UFG titanium enhances its biological response while maintaining corrosion resistance, supporting its clinical use in long-term dental and orthopedic implants with integrated drug-release functionality.

## 1. Introduction

Since approximately 500 BC, when primitive gold application was first used for tooth replacement by the Etruscans, materials for implantology have undergone considerable development [1]. After titanium fixators were first tested in 1978, the material itself became very popular for the construction and manufacturing of long-term and rather permanent applications for hard tissue replacement [2]. The main alloy used is so-called commercially pure titanium (CP Ti). This metal is available in four grades, numbered 1 to 4, according to its purity and interstitial atom content [3]. These grades may differ in corrosion and mechanical properties due to impurities that are likely bound with titanium or otherwise form secondary phases in a titanium matrix. Particles of these phases hinder the dislocation movement on slip planes, which causes precipitation strengthening of the matrix. On the other hand, under certain conditions, they decrease corrosion resistance by the spontaneous initiation of micro-sized corrosion cells in specific environments [3,4,5].

As the ultimate tensile strength of CP Ti is usually lower than that of austenitic stainless steels (240–550 MPa for grades 1–4, respectively), Ti6Al4V was introduced as CP Ti grade 5, which shows ultimate tensile strength (UTS) above 850 MPa [6]. The microstructure of this alloy is formed by a mixture of HCP α and BCC β phases. Due to higher mechanical properties, CP Ti grade 5 is commonly applied for highly loaded implants, where other titanium alloys fail due to overload or fatigue mechanisms [7]. Later on, some progressive or experimental multicomponent alloys with a two-phase microstructure (e.g., Ti6Al7Nb [8], Ti13Nb13Zr [9], Ti29Nb13Ta5Zr [10], etc.) were introduced and are currently being validated for practical use in the field of implantology. Although these progressive titanium alloys mostly have promising mechanical properties, their corrosion resistance is fundamentally limited by precipitates in the matrix [11]. Particles of secondary phases dispersed in the microstructure degrade corrosion resistance significantly. Furthermore, the elements released into the environment during the active corrosion process of implants may cause secondary body reactions [12].

Another way of improving the mechanical properties of materials is significant grain refinement by intense thermomechanical treatment with limited recrystallization. This was also shown as a promising way for titanium, when CP Ti, after a severe plastic deformation (SPD) process, reaches UTS up to 1240 MPa and yield strength (YS) up to 1200 MPa [13,14]. The corrosion properties of ultrafine grain (UFG) CP Ti are not usually influenced distinctly by the SPD process and remain stable, as there are no secondary phase particles present in the microstructure [15]. Due to the larger area of grain boundaries per unit volume, there exists a massive accumulation of grain boundary surface energy in the microstructure [16]. Consequently, the grain boundaries are more reactive (become less noble) than grain interiors (acting more noble), which makes grain boundaries sensitive to intercrystalline corrosion as a result of the formation of localized submicron corrosion cells [17].

Titanium-based materials are well known for their high potential for hard tissue bonding as discovered during the first tests in 1978 [18]. This is mostly caused by the high stability of physicochemical interactions between titanium dioxide (TiO_2_) films, spontaneously formed on oxygen-exposed surfaces, and amino acids contained in different tissues [19] or blood serum [18]. The interaction between the material and the surrounding tissue can be subsequently enhanced by coating of the active surface by bioactive components like hydroxyapatite or calcium phosphate-based minerals, leaving the final surface highly porous and with increased roughness [20]. Other coating processes include composite coatings, titanium nitride coatings, carbon, glass, and ceramic coatings, as well as TiO_2_ film coatings [21]. The disadvantage of coating processes is the need for elevated temperature to achieve strong bonding between the coating and the substrate material. Unfortunately, the high temperature may result in microstructure degradation, especially when applied to UFG titanium, where spontaneous recrystallization occurs, leading to grain coarsening and a decrease in mechanical properties [22]. To minimize this risk, chemical or even electrochemical surface treatments can be applied. Because the titanium-based materials are considered highly chemically resistant, aggressive solutions containing a concentrated mixture of an oxidizer and specific corrosion agents must be applied, leaving the surface covered with wide but shallow unorganized corrosion pits [23]. Less concentrated solutions can be used in the electrochemical process called anodization, where the chemical oxidizer is substituted by an external source of electric current. Highly organized formation of TiO_2_ nanopores or even nanotubes can be obtained during this process; furthermore, the geometry of the formation can be actively changed by anodization voltage, and the layer thickness is determined by the passed electric charge, i.e., time of anodization [15]. Chemical treatment of alkalized titanium surfaces by direct immersion in alkaline solutions leaves the surface significantly active with higher values of wettability [24,25]. On the other hand, the negative effect of defects localized in the anodized TiO_2_ layer can be partially reduced by passivation in oxidizing acids [26]. The anodized layer exhibits a large free volume, which can be intentionally saturated by active chemicals and, if applied as an implant, serves as a targeted drug delivery system [27]. Unless this possibility had been studied in the past [28,29], the synergistic combination of ultrafine-grained (UFG) titanium, which provides improved mechanical properties, and its surface modification by anodization to form nanotextured TiO_2_ layers capable of hosting bioactive substances, remains insufficiently explored. This study presents a comprehensive evaluation of the anodization process applied to commercially pure titanium with both conventional and ultrafine-grain structures, with an emphasis on electrochemical and biological performance. The aim of this work was to assess how anodization parameters (20, 40, and 60 V) influence surface morphology, corrosion resistance, wettability, and cytocompatibility of UFG titanium and to verify its suitability for application in Nanoimplant^®^ prototypes.

Although anodized titanium has been studied extensively, most investigations have focused on materials with conventional grain sizes. The combined effects of the UFG microstructure and electrochemical oxidation on corrosion behavior, surface energy, cell response, and local drug delivery potential have not yet been fully understood. In addition, very few studies address the technological transfer of anodization to real miniature implant geometries with clinically relevant shapes and surface areas.

Based on these gaps, the aim of this study was to verify that anodization of UFG titanium would generate uniform and mechanically stable TiO_2_ layers with enhanced biological interaction and an increased ability to intercalate small therapeutic molecules, without compromising corrosion resistance or mechanical integrity.

## 2. Materials and Methods

### 2.1. Material and Its Processing Parameters

Investigations were mainly performed on CP titanium grade 4 with ultrafine grain size. This UFG CP Ti was obtained using SPD processing by equal channel angular pressing (ECAP) conducted at 300 °C with 8 passes via the route B_C_ [14]. Especially for the purpose of corrosion parameter comparison, there was also CP Ti grade 3 with conventional grain size included in the selected tests. The chemical composition and basic mechanical properties of both grades are summarized in Table 1. The mechanical properties of ECAP CP Ti grade 4 were determined by tensile tests; all other values are taken from the ASTM B265 [30].

**Figure 1:**
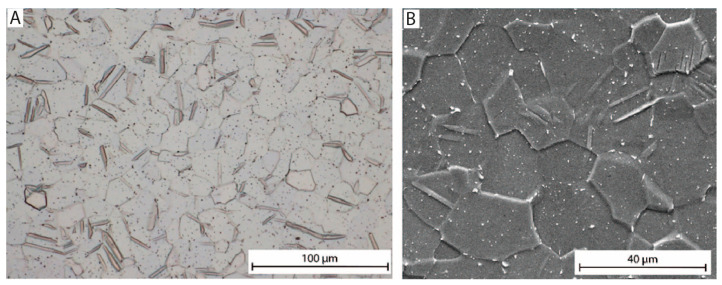
Microstructure of the reference sample (CP-Ti gr. 3) observed by (**A**) light and (**B**) scanning electron microscopy image in SE mode.

The microstructure of CP Ti grade 3, revealed by light microscopy (LM) after mechanical polishing, followed by electrochemical polishing (30 V/10 s in Struers A3 solution), is shown in Figure 1. It is obvious that the presented microstructure with equiaxed grains is typical for material after a hot forming process with spontaneous primary recrystallization. The average grain size is approximately 30 μm.

**Figure 2:**
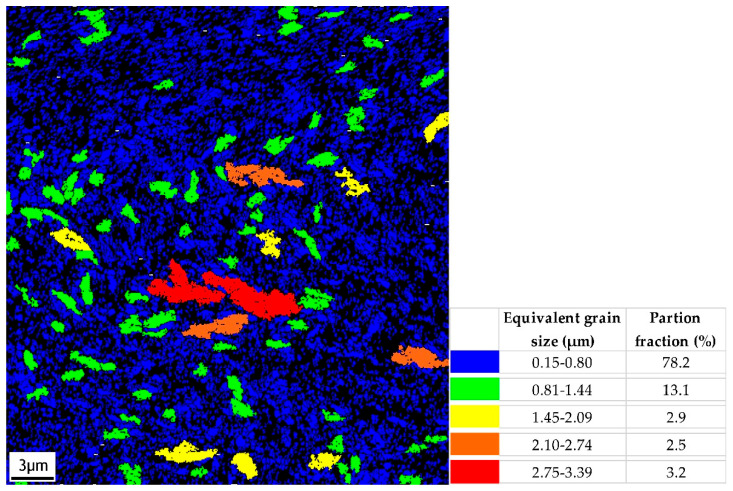
Grain size map, EBSD, sample UFG CP Ti grade 4, cross-section.

**Figure 3:**
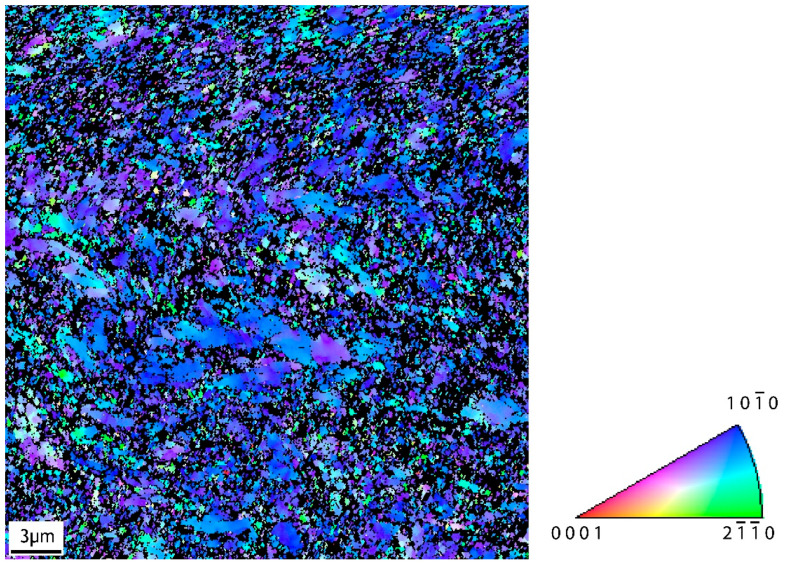
IPF orientation map for the LD, sample UFG CP Ti grade 4, cross-section.

Figure 2 illustrates a grain size map of CP Ti grade 4 after ECAP processing obtained by the electron back-scattered diffraction (EBSD) technique in a scanning electron microscope (SEM-FEI QUANTA FEG 450+, Brno, Czech Republic). The size of individual grains was evaluated as the equivalent grain diameter, which was defined as the diameter of a circle with the same area. The minimum grain size, which could be determined for the measurement conditions used, was 0.15 µm. Figure 3 shows an IPF orientation map for the LD (longitudinal direction during ECAP). The IPF map reveals a pronounced preferred orientation of grains. The orientation of most grains in the LD is close to {10$\bar{1}$0}.

**Figure 4:**
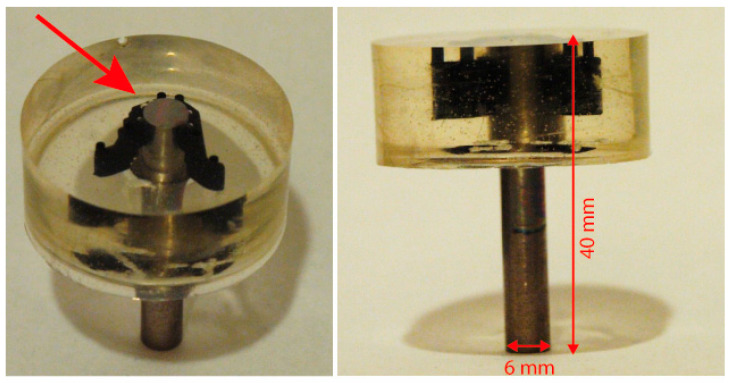
Samples after mounting in resin; an arrow marks the cross-section area for testing.

For the purpose of electrochemical oxidation, each material was machined into cylindrical shape, ϕ6 × 40 mm. Samples were partially mounted into bisphenol transparent resin (Specifix by Struers). Orientation of the rods in mounted samples (see Figure 4) allowed mechanical preparation and electrochemical treatment on rods’ cross-sections. The investigated sections also remained constant during corrosion tests, where the risk of crevice corrosion under an O-ring spacer was completely eliminated [31].

### 2.2. Electrochemical Surface Oxidation

**Figure 5:**
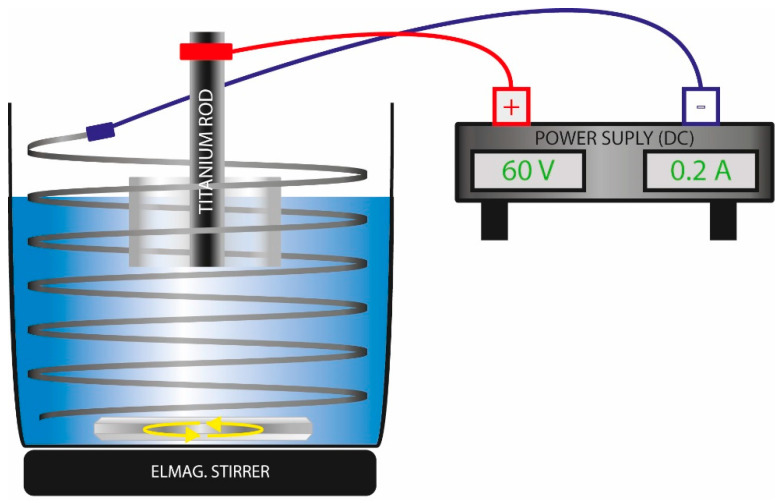
Schema of anodization apparatus.

After mechanical grinding (SiC papers up to #4000) and polishing with colloidal Al_2_O_3_ suspension (Struers), the cross-section surfaces of the rods were ultrasonically cleaned and degreased in pure ethanol for 10 min. The samples were then removed from the bath and allowed to dry completely to ensure full evaporation of residual ethanol. For anodization, the samples were fixed in a laboratory holder and partially immersed in an electrolyte composed of 79 wt.% ethylene glycol, 20 wt.% deionized water, and 1 wt.% NH_4_F (all reagents from VWR™). A platinized titanium wire, coiled around the sample, was placed in the solution to ensure a homogeneous electric field distribution. The sample and the platinized wire were connected to the positive and negative terminals of a DC power supply with adjustable voltage using crocodile clips. A schematic representation of the anodization setup is shown in Figure 5.

**Figure 6:**
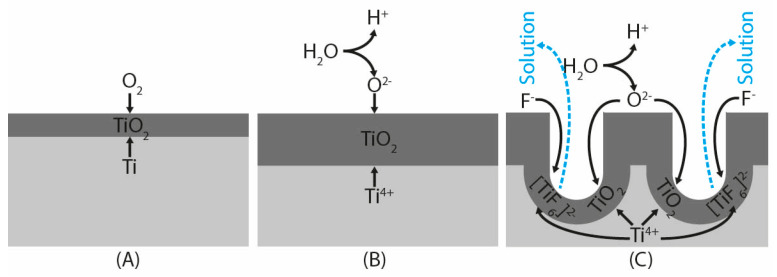
(**A**) Spontaneous formation of passive oxide layer; (**B**) accelerated electrochemical oxidation related to electrolysis as a source of oxygen; (**C**) selective dissolution of oxide layer during accelerated oxidation in fluorine-rich solutions.

In general, the mechanism of the TiO_2_ porous layer formation on a clean titanium surface (Figure 6A) in fluorine-ion-based electrolytes is said to occur as a result of three simultaneous processes: the field-assisted oxidation of Ti metal to form titanium dioxide (Figure 6B and Equation (1)), the field-assisted dissolution of Ti metal ions in the electrolyte, more precisely the chemical dissolution of TiO_2_ due to etching by fluoride ions (Figure 6C and Equation (3)), which is enhanced by the presence of H^+^ ions produced by electrochemical water molecules splitting when sufficient external voltage is applied (Equation (2)) [32,33]. This process leaves the surface covered either by nanopores [34] or thin-walled nanotubes [27,35]. As the oxygen bubbles were generated on the anodized surface, to prevent surface/solution contact, electromagnetic stirring was applied at 180 RPM to eliminate them. Anodization time was set to 300 s; according to previous tests and measurements, longer times of anodization led to thicker anodized layers with lower adhesion to the UFG CP Ti substrate.

(1) $$Ti+O_{2}\to {TiO}_{2}$$

(2) $$H_{2}O\to O_{2}+{4\;e}^{-}+{4\;H}^{+}$$

(3) $$TiO_{2}+6\;F^{-}+\;4\;H^{+}\to \;\;({TiF_{6})}^{-2}+2\;H_{2}O\;$$

Three sets of UFG CP Ti samples were anodized at different voltages for the purpose of the present study. The anodization voltages of 20, 40, and 60 V were selected based on previous optimization studies [36,37], where these values generated TiO_2_ layers with distinct nanotubular to nanoporous morphologies and stable adhesion to titanium substrates. Each experimental condition was performed on three independent specimens, and all electrochemical, wettability, and biological tests were conducted in triplicate to ensure reproducibility. A sample of CP Ti grade 3 was not anodized and was used as a reference to the anodized ones. The parameters of processing for each sample are listed in Table 2.

### 2.3. Corrosion Tests

Given the substantial corrosion resistance inherent in titanium-based materials, conducting direct immersion corrosion trials would prove inefficient in terms of time consumption. Consequently, the potentiodynamic polarization technique was employed to ascertain specific corrosion characteristics of the materials under examination. The testing parameters adhered to the guidelines established by ASTM F 2129 and ASTM F 746 [38,39], albeit with certain alterations to account for the unique specifications of the samples. Corrosion cells, featuring a reduced exposure aperture and a total volume of 20 mL, were utilized for these assays. The surface area subjected to testing remained consistent and was derived from the measured diameter of the rod specimen. Testing was conducted within an isotonic saline solution (comprising 0.9 wt.% NaCl dissolved in deionized H_2_O) explicitly designed to mimic the biological environment of living tissue. No gas purging was implemented throughout the experiments, and the temperature was maintained precisely at 25 °C. A standard three-electrode configuration was implemented: the sample served as the working electrode, a saturated calomel electrode (SCE, measuring +241 mV relative to SHE) [40] functioned as the reference electrode, and a high-purity carbon rod acted as the counter (auxiliary) electrode.

A stabilization period totaling 90 min was enforced subsequent to filling the corrosion cells with the solution to allow partial corrosion reactions to settle [41]. Prior to initiating the potentiodynamic sweep, which was executed using a Voltalab PGZ 100 potentiostat (Radiometer Analytical, Copenhagen, Denmark), the starting potential was established at −100 mV relative to the established open-circuit potential (OCP) following the achievement of corrosion equilibrium, with the polarization scan rate set to 60 mV.min^−1^. The measured current traversing the sample, in relation to the potential applied to it, was continuously recorded during the measurement phase. The potential was incrementally imposed upon the specimen, escalating steadily as a function of time according to the defined polarization rate. This entire process was managed and controlled via the Voltamaster 10 software package.

### 2.4. Surface Wettability Test

The wettability of the tested surfaces was evaluated using the sessile drop method, which enables direct measurement of the static contact angle and assessment of the surface’s affinity toward liquids. Measurements were performed using the SEE contact angle system, and the surface free energy was subsequently calculated with Advex Instrument software, version E (Brno, Czech Republic). Droplets of deionized water (2 µL each) were gently deposited onto the examined surfaces, and the contact angle (θ) was obtained by fitting the tangent at the point of intersection between the liquid, solid, and vapor phases [42].

The surface free energy of the solid was derived from Young’s equation (Equation (4)), in which γ_SV_, γ_LV_, and γ_SL_ denote the interfacial tensions at the solid–vapor, liquid–vapor, and solid–liquid boundaries, respectively [43].

(4) $$\gamma_{sv}-\gamma_{sl}=\gamma_{lv}*\;cos\;\theta$$

### 2.5. Drug Intercalation and Fourier-Transform Infrared Spectroscopy (FTIR) Characterization

**Figure 7:**
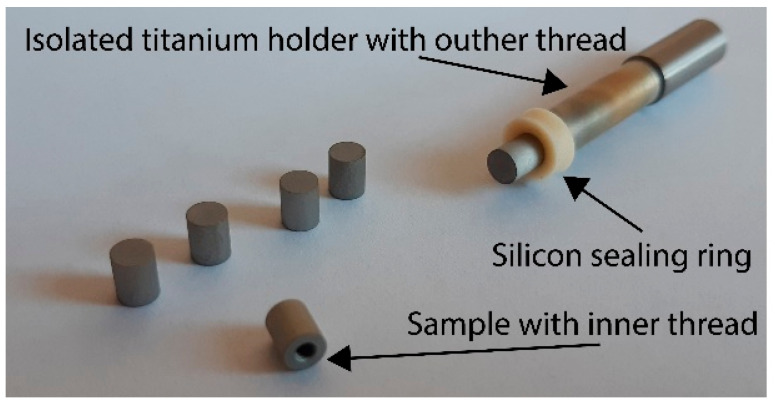
Schema of samples used for intercalation followed by FTIR characterization.

As per test requirements, a smaller sample (i.e., 5 mm in length and diameter) had to be manufactured in order to completely suit the test chamber of the spectrometer. These samples were attached to a threaded titanium holder with an isolated surface, as illustrated in Figure 7, and one after another was anodized under the same conditions as the samples for the corrosion tests. There were two different solutions used for anodized surface intercalation. Ibuprofen 1% aqueous solution was obtained from Magistraliter Pharma Company and used as prepared. For the preparation of standard solutions for high-performance liquid chromatography (HPLC) measurements, ibuprofen (Sigma-Aldrich, Saint Louis, MO, USA, 99%) was used. There was an anhydrous dimethyl sulfoxide (Sigma-Aldrich, ≥99.9%) used as a second intercalation solution. There were samples anodized at 60 V selected for intercalation according to the results of previous corrosion and wettability tests. Anodized samples were placed in the Eppendorf flasks, and 2 mL of intercalation solutions were added to them. The holder with the Eppendorf flasks was stored in the dark for 24 h to prevent any interaction of anodized surfaces and solutions, causing decomposition of its active substances due to the possible photo-catalytic effect of nanostructured titanium surfaces [44]. After the intercalation process, the samples were removed from the Eppendorf flasks, repeatedly cleaned by high-purity distilled water, air-dried, and immediately characterized.

Fourier-transform infrared spectroscopy (FTIR) was used for the determination of surface adsorption capability. FTIR spectra were recorded on a Nicolet iS50 spectrometer (Thermo Fisher Scientific, Madison, WI, USA) using the single-reflection mode on a diamond crystal (32 scans, 4 cm^−1^ resolution). Background (ambient air) measurement and standard instrument-based polynomic baseline correction were used to record and interpret the spectra. FTIR spectra were obtained on a Nicolet iS50 spectrometer using the single-reflection mode on a diamond crystal (32 scans, 4 cm^−1^ resolution). The measurements were performed in the field of mid-infrared spectroscopy in the spectral range from 2.5 μm to 20 μm (from 4000 cm^−1^ to 400 cm^−1^).

### 2.6. Cell Adhesion and Proliferation

Human osteoblast-like cells (MG-63, ECACC 86051601; Sigma Aldrich, St. Louis, MO, USA) were cultured in Dulbecco’s Modified Eagle Medium (DMEM; Biosera Europe, Nuaille, France) supplemented with 10% (*v*/*v*) fetal bovine serum (FBS; Biosera Europe), 100 U/mL penicillin, 100 µg/mL streptomycin (PAA Laboratories GmbH, Austria), and 2.5 mM L-glutamine (Diagnovum GmbH, Ebsdorfergrund, Germany). Cells were maintained at 37 °C in a humidified atmosphere containing 5% CO_2_. Culture media were replenished regularly to maintain optimal growth conditions. The MG-63 cell line was selected because it is a widely accepted and standardized in vitro model for evaluating cell–material interactions on metallic implant surfaces. It provides reproducible proliferation dynamics and is highly responsive to surface chemistry and morphology, making it suitable for assessing the cytocompatibility of anodized titanium. The use of a single osteoblast-like cell line also minimized biological variability. However, to approximate in vivo conditions more closely, future studies will include primary osteoblasts and co-culture systems. Cell adhesion (2 h after seeding) and proliferation (48 h after seeding) were quantified using the Cell Counting Kit-8 (CCK-8; Bimake, Munich, Germany), following the manufacturer’s protocol with minor volume adjustments. The experimental procedure was adapted from previous work [45]. Cells were seeded at a density of 2.5 × 10^5^ cells/mL onto the tested surfaces.

After 2 h, non-adherent cells were removed by washing with phosphate-buffered saline (PBS), and 550 µL of CCK-8 working solution (50 µL CCK-8 reagent + 500 µL culture medium) was added. After incubation for 16 h at 37 °C, 110 µL aliquots were transferred to a 96-well plate, and absorbance was measured at 450 nm using a Synergy H1 microplate reader (Biotek, Winooski, VT, USA). After media replacement, incubation continued. At 48 h post-seeding, 50 µL of CCK-8 reagent was added directly to each well of the 48-well plate, followed by another 16 h incubation. Absorbance was again recorded at 450 nm. Adhesion and proliferation were expressed relative to the positive control (cells grown on tissue culture polystyrene).

For fluorescence visualization, cells were stained with CellTracker™ Green (Molecular Probes, Eugene, OR, USA) and NucBlue^®^ Live ReadyProbes^®^ Reagent (Hoechst 33342; Life Technologies, Eugene, OR, USA), following the procedure described previously [46]. Briefly, 2 drops of NucBlue reagent were added per 1 mL of culture medium and incubated for 30 min at 37 °C. The medium was then replaced with Live Cell Imaging Solution (Life Technologies). Fluorescent imaging was performed using an Olympus CKX41 inverted microscope (Olympus, Hamburg, Germany) at 400× magnification. In a complementary assay, cells were stained with Crystal Violet (Sigma Aldrich). After PBS washing, cells were fixed with 2.5% glutaraldehyde in PBS (pH 6.7–7.1) for 30 min at room temperature, rinsed again with PBS, and subsequently stained with 0.5% Crystal Violet for 20 min. Excess dye was removed, and samples were washed three times in deionized water. After drying, samples were observed and imaged using a Leica S9i stereomicroscope (Leica Microsystems, Wetzlar, Germany). Statistical analysis was conducted using a two-tailed Student’s *t*-test. Differences were considered statistically significant at *p* < 0.05.

### 2.7. Practical Adhesion and Tribological Test

**Figure 8:**
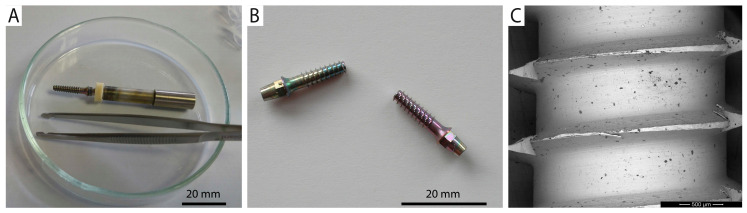
(**A**) Nanoimplant^®^ screwed on holder before anodization; (**B**) Nanoimplant^®^ after anodization at 40 V-left and 60 V-right; (**C**) topography of Nanoimplant^®^ surface after anodization obtained by SEM in back-scattered electron (BSE) mode.

Unlike the previous tests, which were performed on ideal dummy samples, this test aims to investigate technology transfer from ideal samples to real implants. UFG CP Ti has been successfully used for teeth implant manufacturing [13,47,48]; therefore, a set of Nanoimplant^®^ [49] was machined from this material. The same holder was used for implant anodization and the protocol of anodization was applied. The samples were rinsed by deionized water and left to air dry completely in the end. Implants before and after anodization are presented in Figure 8.

Conditions of the tests were selected intentionally to simulate the real process of implantation into bone. According to previous research, three different materials were used to simulate hard tissue structures of a human bone—artificial bone model [50], dried ash wood [51,52], and fresh cow rib [53,54]. The same procedure was performed as recommended for real implantation [55,56]. The only difference was that immediately after application, the implants were extracted and rinsed with deionized water to clean possible loose residues of testing material, especially tissue of cow rib. After these tests, the surfaces of extracted implants were studied by SEM to evaluate the real damage of anodized surfaces.

## 3. Results

### 3.1. Surface Morphology

**Figure 9:**
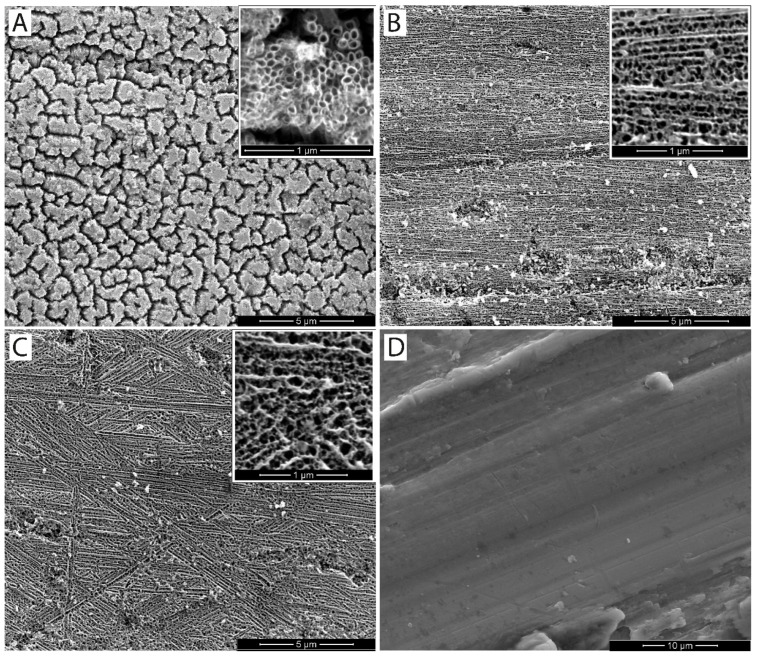
Surface morphology of the UFG CP Ti grade 4 anodized at (**A**) 20 V/300 s, (**B**) 40 V/300 s, and (**C**) 60 V/300 s, and (**D**) surface morphology of the reference sample CP Ti grade 3 after polishing; SEM images in SE mode.

After anodization, all samples were rinsed thoroughly with deionized water and dried in a desiccator at room temperature. The non-anodized CP Ti reference sample was only cleaned with acetone after final polishing and required no additional treatment. The surface morphology of anodized samples (Figure 9A–C) and the reference surface (Figure 9D) was examined using SEM in secondary electron (SE) mode.

The application of anodization completely altered the surface morphology when compared to the mechanically polished reference material (Figure 9D), where only shallow grinding and polishing scratches were visible. At 20 V (Figure 9A), the oxide layer consisted of partially separated clusters of vertically aligned TiO_2_ nanotubes with an average diameter of approximately 50 nm. The enlarged inset in Figure 9A shows these nanotube bundles more clearly. Their “island-like” appearance is caused by local stress accumulation during oxide growth and slight crystallographic misorientation between underlying ultrafine grains, which leads to the partial separation of nanotube domains. This phenomenon is typical for anodized UFG titanium and has been described as stress-induced clustering of nanotubes [25].

At higher voltages (40 V and 60 V; Figure 9B,C), anodization produced a more homogeneous and compact nanoporous TiO_2_ layer. In these samples, the initial surface topography is still preserved—the shallow polishing scratches remain visible—but the oxide is more uniform, as the higher electric field promotes field-assisted dissolution and suppresses the formation of separated nanotube bundles. The pore size increases with voltage, and no pronounced island-like defects are observed.

### 3.2. Potentiodynamic Corrosion Test

After the time lag, when equilibrium between oxidation and reduction reactions was set [57], corrosion cells were connected to a Voltalab PGZ 100 potentiostat, and the open-circuit potential (OCP) was measured for 60 s. As the OCP remained very stable (its shift was <1 mV/min in all cases), the polarization sequence was initialized.

During the first polarization, stage transition between the cathodic and anodic states was recorded. Current density decreased to zero value and increased again. In this so-called Tafel region, a polarization curve exhibits a V-like shape, if a semilogarithmic plot is used for its representation [58]. This part of the curve was also used for the determination of corrosion potential (E_cor_), polarization resistance (R_p_), and corrosion current density (J_c_) by Tafel extrapolation [59]. There was an exchange of four electrons during corrosion reactions (Ti^0^→Ti^4+^) [60]; an average material molar mass of 47.9 g·mol^−1^ [61] and a density of 4.5 g·cm^−3^ [62] were considered for calculating the corrosion rate from current density according to [63]. For the control, the Stern–Geary relation [64] was used for the determination of corrosion potential and polarization resistance as well. The results of both methods should be comparable. Potentiodynamic polarization measurements were performed in triplicate for each surface, and all mentioned parameters were determined automatically by Voltamaster 10. Results (mean values and standard deviations) are listed in Table 3.

**Figure 10:**
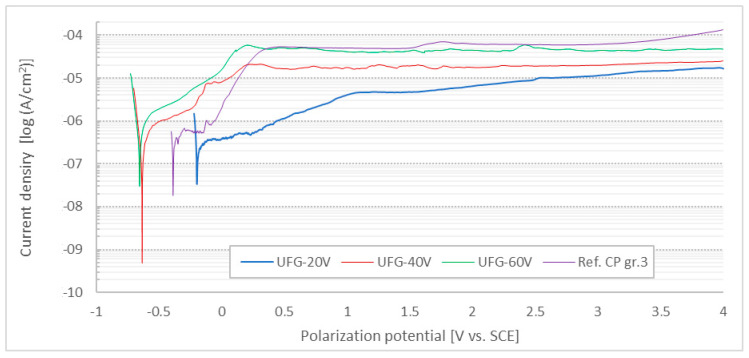
Polarization curves of samples (a typical curve for each sample was selected for presentation).

When the Tafel region was exceeded, the samples were subsequently polarized to higher potentials to evaluate the anticorrosion effect of anodically formed layers against pitting evolution on the tested surfaces. The procedure was stopped automatically when 4000 mV vs. SCE was reached. Typical corrosion curves in the semilogarithmic plot are presented in Figure 10.

According to the Tafel extrapolation results, increasing the anodization voltage led to a progressive shift in the corrosion potential towards less noble values. Higher voltages also resulted in an increased corrosion rate and a noticeable decrease in the polarization resistance, indicating a reduced protective efficiency of the anodic oxide layer.

The corrosion parameters of the reference CP Ti sample were comparable to those of the anodized UFG samples; however, the reference surface exhibited the lowest corrosion rate and therefore the most favorable corrosion performance. No sudden rises in current density were detected on the polarization curves, suggesting that pitting corrosion was not initiated and reverse scanning was not required, as all surfaces remained in the passive state throughout the experiment. Although the polarization curves did not show a clear transition into the transpassive region, the gradual increase in current density at higher potentials may indicate the onset of electrochemical degradation of the oxide layer through anodic reactions [65]. Hence, all surfaces were observed by SEM, but no signs of localized corrosion damage were found at all.

### 3.3. Surface Wettability

**Figure 11:**
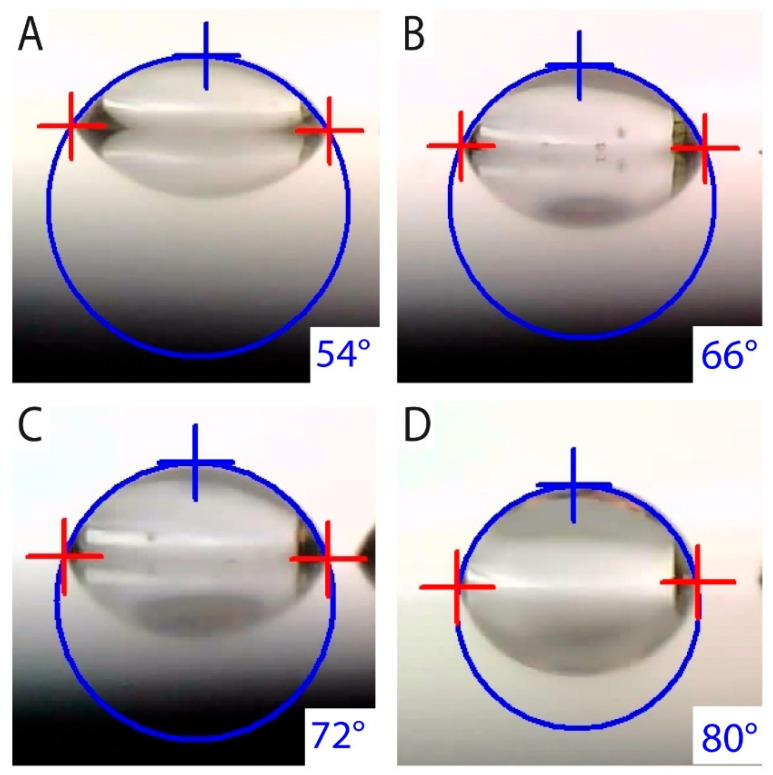
Typical droplets on the surface of samples (**A**) UFG-20V, (**B**) UFG-40, (**C**) UFG-60V, and (**D**) reference sample CP grade 3.

All surfaces were fine-polished before anodization to minimize the risk of results being affected by inconsistent surface roughness. Before the test, all samples were cleaned separately in an ultrasonic acetone bath with testing surfaces facing up to avoid being scratched. The contact angles of 10 droplets were measured for each sample. Mean values together with corresponding standard deviations are presented in Table 4. Due to the Cosine function used to calculate surface energy, its standard deviation is not symmetrical; therefore, the value was averaged too. Figure 11 illustrates images of captured droplets together with their shape extrapolation according to [66].

The polished reference sample exhibited the highest contact angle (81°), indicating the lowest surface wettability. In contrast, all anodized surfaces showed increased hydrophilicity, with contact angles ranging from 52° to 71°. The standard deviations of contact angle measurements were below 3°, and the calculated surface energy values showed deviations of less than 2 mJ·m^−2^, confirming good measurement reproducibility.

### 3.4. Practical Adhesion and Tribological Test

According to previous test results, only the most promising anodization protocol was used for application to Nanoimplant^®^ samples, i.e., 60 V for 300 s. Considering the surface pore size, the layer anodized at a higher voltage can be saturated by specific substances more effectively than the layer with narrow pores formed at 20 V [67].

**Figure 12:**
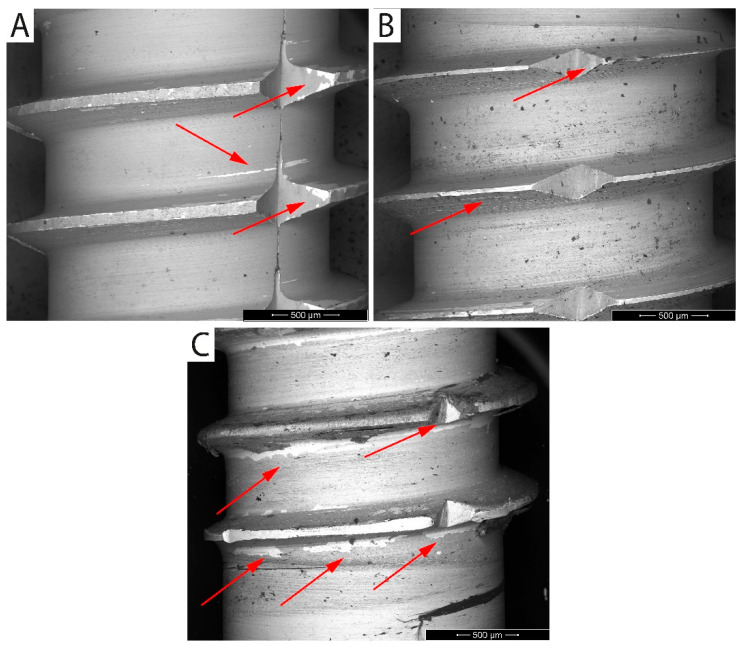
Implants’ surfaces after their extraction from (**A**) artificial bone model, (**B**) dried ash wood, and (**C**) fresh cow rib; SEM images in BSE mode (material contrast).

Before the SEM observation, the samples were immersed in an ultrasonic bath with demineralized water to clean the surface of organic residues for 30 s. BSE mode was used for the surface inspection of extracted implants. Due to the material contrast, areas of the anodized layer delamination were clearly visible as they appeared brighter [68]. The surfaces of extracted implants are shown in Figure 12, where arrows point to areas of the damaged anodized layer.

The UFG CP Ti samples showed different levels of surface damage. The implant surface extracted from dried ash wood showed only minor surface damage. The surfaces of implants extracted from both the artificial bone model and fresh cow rib were damaged, especially along threads, while anodized surfaces among threads remained intact. The examination of implants was difficult due to organic impurities bonded to the surface, especially after extraction from fresh cow rib.

### 3.5. Layer Intercalation and FTIR

**Figure 13:**
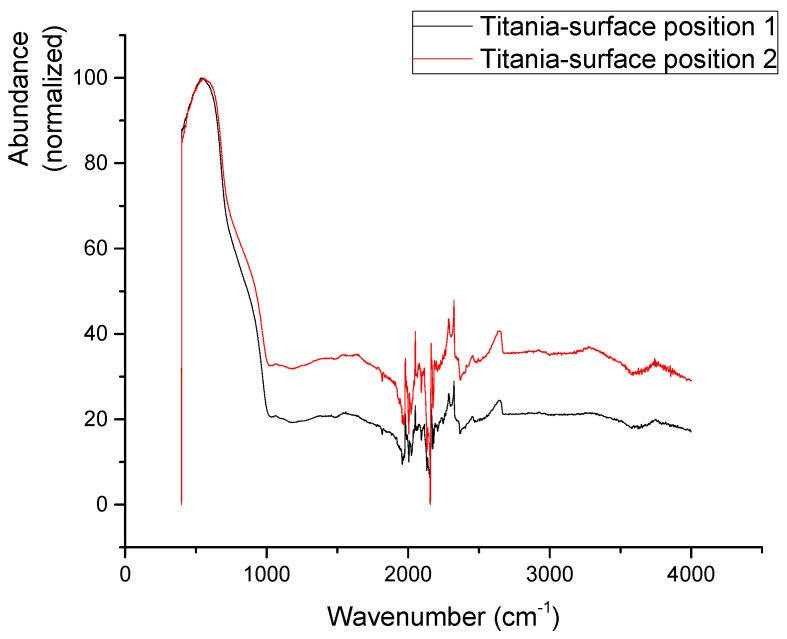
Infrared spectra of the anodized surface from two spots of the same sample.

FTIR spectra of a pristine anodized surface (Figure 13) are dominated by a broad absorption peak around 650 cm^−1^ with a clear shoulder around 860 cm^−1^. This corresponds well to ν(Ti-O) vibrations and δ(Ti-O-Ti) vibrations in titania nanotubes [69]. A less visible broad peak around 3000 cm^−1^ suggests that some O-H bonds in the form of –OH termination of the TiO_2_ structure or in the form of adsorbed water are also present. The area 2000–2600 cm^−1^ of the spectra is noised as CO_2_ and other aerial impurities, together with the residual signal of the FTIR crystal, influence the spectrum [70].

**Figure 14:**
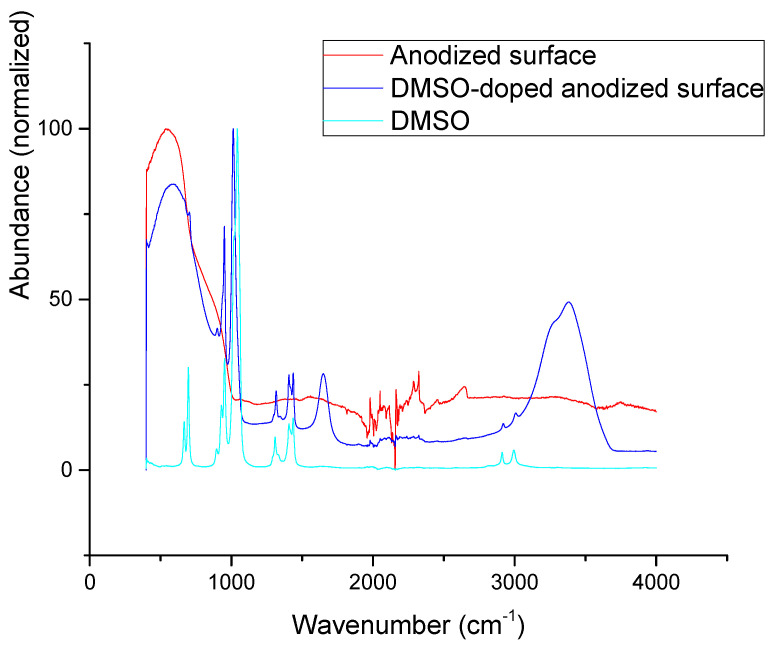
Infrared spectra of anodized surfaces, DMSO-doped anodized surface, and DMSO.

DMSO-doped titania surface shows (Figure 14) most of the major peaks of DMSO: ν(C-H): 2995 cm^−1^ and 2910 cm^−1^, *δ*(CH_3_): 1435 cm^−1^, 1405 cm^−1^, and 1310 cm^−1^, and ν(S=O): 1010 cm^−1^. Further DMSO vibrations are also visible in the spectrum, such as ν(C-S) 665 cm^−1^ and 695 cm^−1^, yet they are partially hindered by strong absorptions of Ti-O bonds. Clearly visible δ(H-O-H) vibration at 1650 cm^−1^ and ν(O-H) 2700–3700 cm^−1^ vibrations suggests a significant presence of water, which is not a surprise as the DMSO is known to be hygroscopic and the surfaces were exposed to aerobic moisture. Only one DMSO vibration diminishes in DMSO-doped samples: ν(S=O) 1045 cm^−1^. No significant changes in Ti-O(-Ti) vibrations can be seen on the doped surfaces. Altogether, a comparison of DMSO and DMSO-doped surface IR spectra suggests that DMSO is mostly physisorbed on the titania surface with O-atom fixed, probably in some form of long-range (CH3)2S=O---Ti interaction.

**Figure 15:**
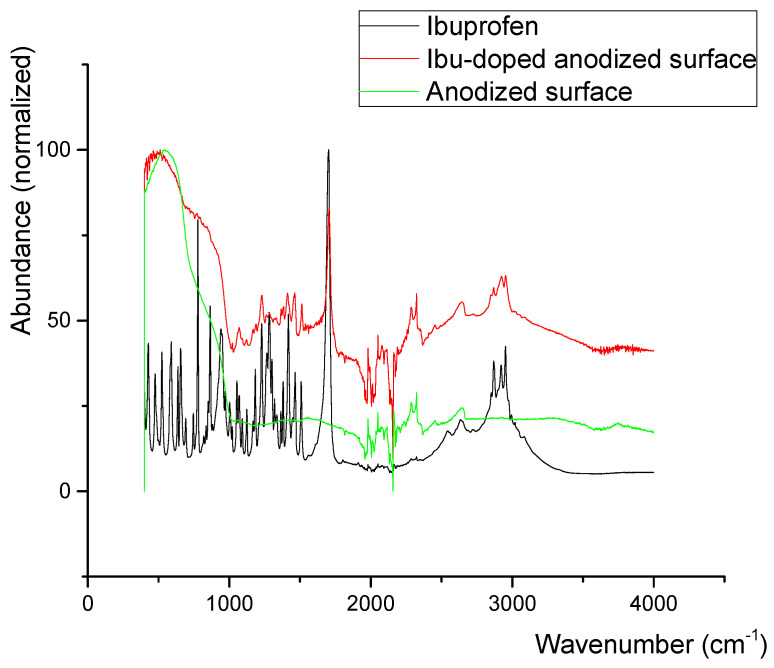
Infrared spectra of pristine anodized surface, Ibu-doped anodized surface, and ibuprofen.

In ibuprofen-doped titania surfaces (Figure 15), the situation is less clear. No significant changes in Ti-O(-Ti) vibration patterns are visible. Traces of ν(C-H) aromatic and ν(C-H) aliphatic signals of ibuprofen can be seen in regions 2800 cm^−1^ to 3100 cm^−1^ along with the characteristic overtone and δ(C(O)-O-H---O) vibrations in the region of 2500 cm^−1^ to 2800 cm^−1^ and with some skeletal vibrations in the region of 1010 cm^−1^ to 1650 cm^−1^ [71]. The signals are, though, always broadened and somewhat shifted. The only signal which can be undoubtedly identified is the signal of the ν(C=O) bond, always slightly shifted from the original wavenumber of ibuprofen (1700 cm^−1^ in ibuprofen vs. 1705 cm^−1^ in doped surface). It can be concluded that ibuprofen is present on the anodized surface. The nature of its adsorption is not clear though. Both physisorption and chemisorption cannot be definitely ruled out.

### 3.6. Cell Adhesion and Proliferation

**Figure 16:**
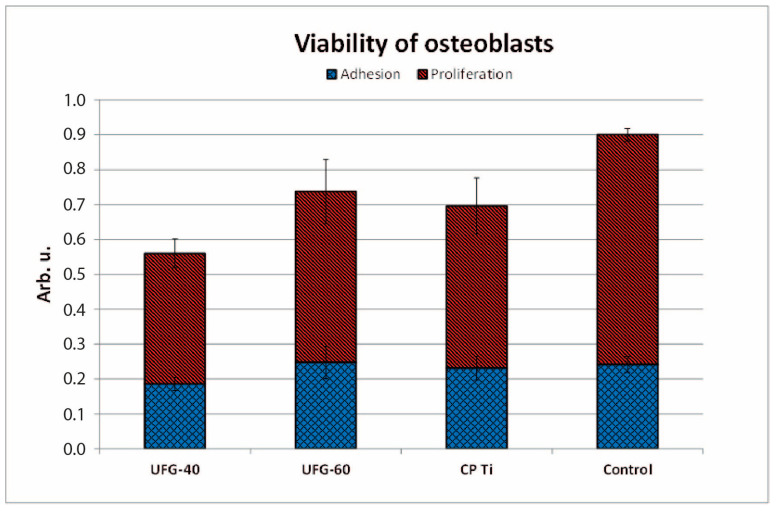
Comparison of cell adhesion and proliferation on the selected surfaces. Error bars indicate standard deviation.

**Figure 17:**
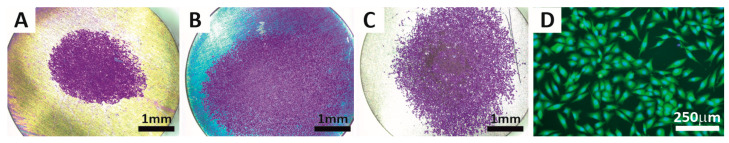
Osteoblasts on the surfaces of samples and control cells after 48 h of proliferation. (**A**) UFG-40 with cells stained by crystal violet, (**B**) UFG-60 with cells stained by crystal violet, (**C**) CP Ti with cells stained by crystal violet, (**D**) cells on the plastic tissue plate stained by NUC Blue and CellTracker.

For biological evaluation, samples anodized at 40 V and 60 V were selected. All tested titanium surfaces were non-toxic to osteoblast cultures. However, significant differences were observed in cell adhesion and proliferation between etched, anodized, and control surfaces. Cell adhesion on UFG-40V was lower by 16% compared to CP Ti (*p* = 0.0444) and by 23% compared to control tissue culture plastic (*p* = 0.0049). In contrast, UFG-60 exhibited adhesion values comparable to both CP Ti and the control surface. Cell proliferation on UFG-40V remained significantly reduced compared to control cells (*p* = 0.0016). In the case of UFG-60V, proliferation was slightly higher than on CP Ti but still lower than on the control plate (*p* = 0.0459). A direct comparison of anodized surfaces showed significantly higher adhesion on UFG-60 than on UFG-40 (*p* = 0.0393). Figure 16 displays mean values and corresponding standard deviations for cell adhesion and proliferation. Corresponding *p*-values for all pairwise comparisons (sample vs. control, UFG vs. CP Ti, UFG vs. UFG) are summarized in Table 5. Representative microscopic images of osteoblast morphology on titanium surfaces and the control plate are shown in Figure 17.

## 4. Discussion

All anodized samples were produced from ultrafine-grained (UFG) CP Ti grade 4, which provides a combination of high mechanical strength known from Ti6Al4V and corrosion resistance typical for lower grades of commercially pure titanium [13]. The anodizing voltages and electrolyte composition were selected according to previous studies, reporting stable nanotubular oxide growth and good surface integrity [27,35,72]. Since the voltages used were above the water decomposition threshold, excessive gas evolution can reduce oxide adhesion by forming interfacial bubbles or blisters [73]. For this reason, the water content in the electrolyte was limited to 20 wt.% to increase coating stability. Lower water content (15–25 wt.%) is also known to promote the formation of more compact and mechanically stable anodic oxide layers [74]. Surface morphology was strongly dependent on the applied voltage. At 20 V, highly ordered and vertically aligned nanotubes were formed (Figure 9A). Their organization into small clusters is associated with internal stresses during oxide growth and the limited migration of ions in UFG material [75]. At higher voltages (40–60 V), the electric field intensity caused partial dissolution and restructuring of the nanotube layer, resulting in less ordered and predominantly nanoporous surfaces (Figure 9B,C), which may enhance interfacial adhesion to the substrate [76]. Increasing voltage also led to larger pore diameters and smoother surface features, as oxide growth induces volume expansion and preferential oxidation at sharp edges [25,77]. These effects reduce surface defects from mechanical polishing and promote more uniform oxide coverage. A notable difference compared to conventional grain size titanium is the formation of nanotube clusters already at 20 V in UFG titanium, while in coarse-grained materials, similar structures only occur at higher voltages or longer anodizing times [78,79]. This can be attributed to grain refinement, higher density of grain boundaries, and local crystallographic orientation, which influence oxide growth direction and residual stress distribution. Samples anodized at 40 and 60 V predominantly showed nanoporous rather than nanotubular morphology, which correlates with reduced fluoride content and increased structural stability of the oxide layer on UFG titanium. Heat treatment of the anodized samples was not performed to avoid possible recrystallization of the UFG substrate, which can occur even below 350 °C [80] and would affect both microstructure and mechanical properties. Potentiodynamic polarization measurements confirmed that the UFG-20V surface exhibited the lowest corrosion rate (1.3 µm·year^−1^), comparable to CP Ti grade 3 (1.2 µm·year^−1^), which is commonly used in long-term hard tissue implants [35]. Samples anodized at higher voltages showed a gradual increase in the corrosion rate (1.7 µm·year^−1^ for UFG-40V and 3.7 µm·year^−1^ for UFG-60V), but all values remained well below the commonly accepted limit of 20 µm·year^−1^ for implantable biomaterials [81]. These results are consistent with earlier studies reporting equal or slightly lower corrosion rates for anodized UFG titanium compared to anodized conventional titanium grades [82,83]. It must be acknowledged that electrochemical parameters were calculated using a geometric surface area, which is valid for non-porous surfaces such as CP Ti. However, anodized surfaces possess a significantly higher real surface area due to porosity, especially in UFG-60V samples. As a result, corrosion current density and derived corrosion rates may be overestimated for highly porous oxides [84,85]. This effect should be considered when comparing anodized and non-anodized samples. Corrosion potential (E_corr) provides useful information on the electrochemical nobility of the surface. The CP Ti reference sample exhibited E_corr of −376 mV vs. SCE, in agreement with previously reported values [86]. Anodization at 20 V shifted the potential to more noble values (−204 mV), while anodization at 40 V and 60 V resulted in significantly less noble potentials (−632 and −652 mV, respectively). This behavior is attributed to differences in surface morphology and oxide structure, as similarly observed in other studies [26,87,88]. The UFG microstructure does not appear to have a dominant effect on the corrosion potential, since the obtained values correspond well with those reported for anodized coarse-grained titanium [35,89]. From a practical viewpoint, the lower corrosion potential of UFG-40V and UFG-60V surfaces indicates a higher risk of galvanic interactions when coupled with more noble materials in multi-material systems (e.g., NiTi, stainless steel, untreated titanium alloys) [90,91,92]. This may be relevant in dental or orthopedic implants where dissimilar materials are used. The improved behavior of UFG-20V samples is related to the compact and uniform nanotubular oxide, which provides an effective barrier against ion exchange and inhibits localized corrosion [93,94]. In contrast, highly porous structures formed at higher voltages increase ionic permeability and reduce polarization resistance due to easier electrolyte penetration through open pores.

During anodic polarization up to 4 V vs. SCE, none of the samples exhibited breakdown potential or passivity loss. According to EN ISO 10993–15 [95], materials that do not reach pitting potential at 1752 mV vs. SCE are considered resistant to pitting corrosion. All anodized surfaces fulfilled this criterion, showing stable current density throughout the test, which indicates persistent passivity and only general corrosion processes [95,96]. Surface wettability is an important parameter influencing initial biological responses, including protein adsorption, cell adhesion, and tissue integration. The contact angle of CP Ti grade 3 was measured as 81.4°, indicating a moderately hydrophobic character. All anodized surfaces became more hydrophilic, with contact angles of 52.3° (UFG-20V), 67.9° (UFG-40V), and 71.0° (UFG-60V). Similar trends were reported for anodized titanium with a conventional grain size [27,35,97], confirming that anodization generally enhances hydrophilicity regardless of grain refinement. The most hydrophilic surface (UFG-20V) exhibited a contact angle significantly below 60°, which is considered favorable for osteoblast adhesion. Previous studies demonstrated that osteoblast attachment is enhanced when the contact angle is between 0° and 60°, whereas fibroblast adhesion is strongest in the 60–80° range [98]. This suggests that anodized UFG titanium, particularly at 20 V, may support strong bonding to bone tissue, whereas untreated titanium might exhibit weaker osseointegration and stronger fibrous encapsulation under load-bearing conditions [99]. Enhanced hydrophilicity is also associated with faster adsorption of blood proteins that initiate the early stages of healing and bone formation [100]. Although the direct influence of the UFG microstructure on wettability is still unclear, the obtained values are consistent with anodized surfaces of conventional titanium. For nanoporous surfaces, contact angles around 65–71° have been reported [101,102], whereas nanotubular structures typically show higher hydrophilicity (50–62°) [103,104], in agreement with the present results. Overall, the observed wettability behavior indicates that anodization at lower voltage (20 V) leads to surface conditions more favorable for osteoblast adhesion and osseointegration, while higher voltage anodization yields less hydrophilic surfaces that may reduce bone–implant bonding but still remain within biocompatible limits.

Adhesion of anodic oxide layers to the substrate is a key factor limiting the practical use of anodized implants [105]. For mechanical stability evaluation, only the UFG-60V surface was selected, since the oxide formed at this voltage is the thickest, most porous, and thus most prone to delamination under load [75,106]. Subsequently, the larger pore size can facilitate drug loading or biomolecule incorporation but may also reduce mechanical integrity. In contrast to standard testing procedures using simple insertion and removal in split media [107,108], a more demanding method was applied. Implants were inserted into artificial bone, dried ash wood, and fresh cow rib using a torque motion, which better simulates real implantation stresses. Only minor surface changes were observed after insertion into artificial bone (Figure 12A), and slightly more after insertion into wood (Figure 12B). The most noticeable surface damage occurred after extraction from cow rib (Figure 12C), particularly around individual threads and sharp cutting edges, where mechanical stress is highest and localized compression and shear forces act on the surface. Despite visible damage, no complete delamination, cracking, or flattening of the anodic layer was observed. This indicates better mechanical resilience compared to conventional grain-size titanium in similar studies, where surface deformation or oxide delamination was more pronounced [109]. The improved stability may be related to the refined grain size of the substrate, which enhances mechanical compatibility between the oxide layer and underlying titanium and reduces interfacial stresses. These results show that even though UFG-60V-anodized surfaces are the most porous and electrochemically less stable among anodized samples, they maintain acceptable mechanical resistance under implantation-like conditions.

Ibuprofen and dimethyl sulfoxide (DMSO) were selected as model compounds to evaluate the ability of anodized UFG titanium to act as a local drug delivery platform. These substances differ significantly in molecular size and polarity—DMSO is a small, highly polar molecule (Mw = 78.13 g·mol^−1^), while ibuprofen is larger, contains an aromatic ring and a carboxyl group, and better represents clinically relevant drugs. Their use allows an assessment of both the loading capacity of the nanoporous/nanotubular structures and the interaction mechanisms between TiO_2_ and organic molecules. DMSO was used to determine whether ultrafine nanopores can accommodate very small molecules or solvents. Due to its low steric hindrance, DMSO provides a lower boundary for molecular uptake. In contrast, ibuprofen represents a bulkier molecule whose adsorption and retention can reveal which functional groups interact with the TiO_2_ surface and whether steric limitations prevent infiltration into nanotubes. This information is essential for predicting which classes of drugs or antibacterial agents could be successfully immobilized on modified titanium surfaces. Previous studies have confirmed the ability of anodized titanium (with conventional microstructure) to incorporate ibuprofen within nanotubular layers [28,29,110]. Our results are in agreement—ibuprofen was successfully intercalated into UFG-anodized surfaces, suggesting that substrate grain refinement does not negatively affect the process. The mechanism of ibuprofen binding and its release kinetics are still under investigation and may depend on electrostatic interactions, hydrogen bonding, or physical entrapment within the nanotubes. Although the exact mechanism remains unclear, no degradation of ibuprofen was observed and its chemical compatibility with anodized TiO_2_ was maintained. Overall, the results support the potential of anodized UFG titanium as a functional platform for local drug delivery, particularly for anti-inflammatory or antibacterial agents, while preserving biocompatibility. The biological response to titanium implants is largely determined by the physicochemical properties of the surface, including oxide composition, topography, surface energy, and chemical reactivity. Bioactive surface modification is a well-established approach to promote osseointegration and to reduce the release of potentially cytotoxic metal ions. The interaction between the material and cells is governed not only by direct cell–implant contact but also by protein adsorption, focal adhesion formation, and cell orientation mechanisms [111]. The produced anodized UFG CP Ti surfaces demonstrated good cytocompatibility, consistent with previous observations on conventional titanium grades. The nanotubular surface morphology obtained at 20 V showed enhanced cell adhesion and proliferation compared to the nanoporous morphology, which can be attributed to its combination of high surface area, hydrophilicity, and appropriate nanotube diameter. These results agree with studies reporting that nanotubular TiO_2_ layers promote osteoblast functions and early bone–implant integration [29,112]. The substrate grain refinement did not negatively affect biological performance, suggesting that the primary factor influencing cell behavior is the morphology and chemistry of the anodic oxide rather than the bulk microstructure. In contrast, surfaces anodized at higher voltages (40–60 V) showed lower proliferation or weaker adhesion, which corresponds to their reduced hydrophilicity and nanoporous rather than nanotubular structure. Nevertheless, all anodized samples remained cytocompatible, confirming that none of the tested surfaces induced cytotoxic effects. Furthermore, anodized nanotube layers are capable of functioning as drug delivery reservoirs, enabling the localized administration of anti-inflammatory, antibacterial, or osteogenic agents [113]. This dual functionality—supporting osseointegration while allowing drug loading—represents a promising benefit of anodized UFG titanium surfaces. Overall, the biological results confirm that combining ultrafine-grained titanium with optimized anodization conditions (especially at lower voltages) leads to surfaces that support osteoblast activity and provide potential for advanced implant functionality.

## 5. Conclusions

This research concerns a multidisciplinary study of anodized layers on CP Ti with an ultrafine-grained (UFG) microstructure. The main findings are as follows:

- Anodization at 20 V produced an oxide layer formed by clusters with typical nanotubular morphology. Anodization at 40 V and 60 V produced homogeneous TiO_2_ layers with nanoporous morphology. The pore size increased with the anodization voltage.
- The morphology of oxide layers on UFG CP Ti anodized at 40 V and 60 V matched the morphology of conventional grain size CP Ti anodized in a solution with a significantly lower water content.
- The corrosion rate of the anodized surfaces was slightly higher than that of the untreated CP Ti surface, but still deep under the limit recommended for implants.
- Corrosion potential of anodized surfaces was slightly increased compared to the untreated CP Ti surface, which increases the risk of their damage in the case of coupled corrosion initiation.
- Anodized layers were not prone to pitting corrosion even if polarized to 4000 mV vs. SCE in a solution containing a medium concentration of chlorine ions.
- Anodization of UFG CP Ti decreased the surface contact angle and, at the same time, increased the surface energy, which promotes easier colonization by hard tissue-forming cells.
- The anodized layer formed at 60 V on UFG CP Ti implants exhibited sufficient resistance to mechanical stress arising during simulated implantation procedures.
- The highly porous TiO_2_ surface produced at 60 V enabled the successful intercalation of DMSO and ibuprofen.
- All tested titanium surfaces were non-cytotoxic to osteoblasts. Cell adhesion and proliferation were slightly reduced on UFG-40V, but slightly enhanced on UFG-60V, when compared to the reference CP Ti. Overall, UFG-60V showed higher cell adhesion than UFG-40V and the reference titanium surface. Most parameters studied in this paper (corrosion, wettability, intercalation, biological) on UFG CP Ti and conventional grain size CP Ti anodized at similar conditions were comparable, and no significant differences were found between them.
- The results presented in this paper prove that specific electrochemical oxidation of miniaturized biomedical implants manufactured from UFG CP Ti had a positive effect on their biological performance and could also serve as a targeted drug delivery system while maintaining their excellent corrosion resistance.

## 6. Patents

Part of the research published in this paper is the subject of utility model 2019–36996 registered on 11th December 2019 by the Industrial Property Office of the Czech Republic.

## Figures and Tables

**Table 1:** Chemical composition and mechanical properties of tested materials.

	Mechanical Properties			Chemical Composition (max. wt.%)				
Grade	Tensile Strength (MPa)	Yield Strength (MPa)	Elongation (%)	N	C	H	Fe	O
CP Ti grade 3	min. 450	min. 380	min. 18	0.3	0.8	0.015	0.3	0.35
ECAP Ti grade 4	1143	864	15.4	0.3	0.8	0.015	0.5	0.4

**Table 2:** Parameters of sample processing.

Sample	Material	Anodization Voltage (V)	Time of Anodization (s)
UFG-20V	UFG CP Ti grade 4	20	300
UFG-40V	UFG CP Ti grade 4	40	300
UFG-60V	UFG CP Ti grade 4	60	300
Reference	CP Ti grade 3	-	-

**Table 3:** Corrosion parameters obtained from the Taffel region of corrosion curves.

	Taffel Method				Stearn–Geary Method	
Sample	Corrosion potential E_cor_ (mV vs. SCE)	Polarization resistance R_p_ (kΩ·cm^2^)	Cor. current density J_C_ (nA·cm^−2^)	Corrosion rate C_R_ (μm·year^−1^)	Corrosion potential E_cor_ (mV vs. SCE)	Polarization resistance R_p_ (kΩ·cm^−2^)
UFG-20V	−204 ± 18	125 ± 9	143 ± 14	1.3 ± 0.15	−211 ± 21	128 ± 10
UFG-40V	−632 ± 28	76 ± 8	184 ± 19	1.7 ± 0.22	−634 ± 31	82 ± 9
UFG-60V	−652 ± 33	41 ± 6	410 ± 48	3.7 ± 0.45	−653 ± 35	48 ± 7
Ref. CP Ti gr.3	−376 ± 16	101 ± 8	137 ± 13	1.2 ± 0.14	−375 ± 17	97 ± 7

**Table 4:** Results of contact angle measurements.

Sample	Contact Angle (°)	Calculated Free Surface Energy (mJ·m^−2^)
UFG-20V	52.3 ± 2.1	53.6 ± 1.3
UFG-40V	67.9 ± 1.4	45.3 ± 0.8
UFG-60V	71.0 ± 1.5	39.5 ± 1.0
Ref. CP gr.3	81.4 ± 2.9	32.6 ± 1.9

**Table 5:** Summary of *p*-values (Student’s *t*-test) for cell adhesion and proliferation from Figure 16.

Comparison	*p* (Adhesion)	Significance	*p* (Proliferation)	Significance
V4 vs. Control	0.0049	**	0.0016	**
V6 vs. Control	0.8411	ns	0.0459	*
CP Ti vs. Control	0.6100	ns	0.0070	**
V4 vs. CP Ti	0.0444	*	0.1585	ns
V6 vs. CP Ti	0.5899	ns	0.6392	ns
V6 vs. V4	0.0393	*	0.1017	ns

* *p* < 0.05, ** *p* < 0.01, ns—non-significant.

## Data Availability

The original data presented in the study are openly available in Zenodo at https://doi.org/10.5281/zenodo.17643764.
